# Metabolomic analysis reveals dynamic changes in secondary metabolites of Sophora japonica L. during flower maturation

**DOI:** 10.3389/fpls.2022.916410

**Published:** 2022-08-04

**Authors:** Ji-Rui Wang, Xu-Hong Song, Long-Yun Li, Si-Jia Gao, Fang-Hong Shang, Xiao-Mei Zhang, Yong Yang

**Affiliations:** ^1^Three Grade Laboratory of Chinese Medicine Chemistry, Chongqing Academy of Chinese Materia Medica, Chongqing, China; ^2^Chongqing Sub-Center of National Resource Center for Chinese Materia Medica, China Academy of Chinese Medical Sciences, Chongqing, China

**Keywords:** Sophora japonica L., flower maturation, differential metabolites, flavonoids, multivariate analysis

## Abstract

*Sophora japonica* L. is widely consumed in China because of its medicinal and nutritional value. Its quality is greatly affected by the accumulation of metabolites, which varies with the stage of flower development. However, changes in the characteristics of the secondary metabolites during flower maturity remain unclear. Ultra-high-performance liquid chromatography coupled with electrospray ionization-triple quadrupole-linear ion trap mass spectrometry (UPLC–ESI–QTRAP–MS/MS) revealed dynamic changes in the secondary metabolites of *S. japonica* during the five flower-maturity stages. We monitored 331 metabolites and screened 164. The differential metabolites showed seven trends during flower maturation, with flavonoids and phenolic acids having the most varied expressions. Flower buds (S2–S3) are rich in flavonoids and are thus suitable for use in high-quality medicine or industrial extraction. Our study provides an empirical basis for the informed harvesting of *S. japonica* based on its mode of utilization.

## Introduction

*Sophora japonica* Linn., also known as the pagoda tree, is a tall perennial tree belonging to the Leguminosae family and is widely cultivated in East Asia, especially China. It is valued for its ornamental, medicinal, and edible properties. In northern China, *S. japonica* is often cultivated as an urban tree to absorb dust and beautify the environment (Yu et al., 2021b). In southern China, *S. japonica* is an economically important plant, and the flower/flower bud (FFB-SJ) is used to extract rutin (Horosanskaia et al., 2017) and produce natural dyes (Chen et al., 2010). FFB-SJ is used in food production and for the treatment of hematochezia, hemorrhoids, uterine hemorrhage, hematemesis, epistaxis, exuberant liver fire, dizziness, and giddiness (Chinese Pharmacopoeia Commission C.P, 2020). As early as the Ming Dynasty (*ca.* 1,406 AD), FFB-SJ consumption was recorded in “Jiu Huang Ben Cao” (Zhu et al., 2015). FFB-SJ is increasingly used in the food industry, including as a preservative in sausages (Tang et al., 2019), additives for color and flavor in rice wine (Yang et al., 2021), components of the fat structure in yogurt (Tang et al., 2020), and as an edible packaging film material (Guo et al., 2020).

FFB-SJ is valued for its beautiful appearance, aromatic smell, unique taste, and excellent healthcare function. Previous pharmacological studies have demonstrated that FFB-SJ ameliorates oxidative stress (Mihaylova and Schalow, 2013), regulates anti-melanin precipitation (Lo et al., 2009), ameliorates diabetes (Park et al., 2009), repairs cells damaged by ultraviolet radiation (Li et al., 2019), and improves prostate hypertrophy (Elberry et al., 2020). All of these medicinal functions are related to the composition of the active ingredients of *S. japonica*. FFB-SJ mainly contains flavonoids (Xie et al., 2014), volatile oils (Yao et al., 2011), and polysaccharides (Li et al., 2019). Flavonoids comprise the largest proportion of bioactive compounds in FFB-SJ and are also the most comprehensively studied (Hendrich et al., 2002; Sun et al., 2007; Xie et al., 2014; Maksimenko et al., 2019). Flavonoids are the material basis for *Sophorae Flos*, used to treat hemorrhage (Sachetto et al., 2018), exuberant liver fires (Wu et al., 2017), and cardiovascular and cerebrovascular diseases (Annapurna et al., 2009)—and are also used as quality control indicators (Chinese Pharmacopoeia Commission C.P, 2020). Flavonoids and phenolic acids are outstanding antioxidants because of their phenolic hydroxyl structures that can bind to free radicals (Gao et al., 2022). Traditionally, flowers and flower buds have been used for this purpose. However, the accumulation of metabolites is affected by flower maturity, and biological activity is further affected by changes in chemical composition. Abudayeh et al. (2015) found that the essential oil components of *S. japonica* were affected by the harvest period and that the volatile oil content was highest in the buds. The lectin activity and protein content of *S. japonica* also decrease as the flower buds mature (Cholak et al., 2016). Our previous study showed that the flavonoid content in *S. japonica* extract varied across the five flower-maturity stages, along with variations in antioxidant and tyrosinase inhibition activities (Wang et al., 2019). Candidate genes involved in anthocyanin biosynthesis in wild-type and mutant-type *S. japonica* during different developmental stages were identified based on transcriptomics by Guo et al. (2022). However, most studies have focused on the activities of specific components, while the composition of secondary metabolites at different developmental stages of *S. japonica* has not been fully elucidated.

Metabolomics, which is characterized by rapid, convenient, and high-throughput data generation, can be used to qualitatively and quantitatively analyze metabolites in whole organisms or cells (Feng et al., 2020) and to determine related metabolic processes (Mohammat et al., 2017; Long et al., 2021). With new analytical technologies and comprehensive compound mass spectrometry databases, metabolomics has become an important tool for studying plant physiology, especially in the study of plant stress resistance (Zhao et al., 2021), flower and fruit development (Yang et al., 2019; Yu et al., 2019), and food quality dynamics (Xiao et al., 2021).

This study aimed to describe the dynamic changes in secondary metabolites in *S. japonica* during the five stages of flower maturation using ultra-high-performance liquid chromatography coupled with electrospray ionization-triple quadrupole-linear ion trap mass spectrometry (UPLC–ESI–QTRAP–MS/MS). Our study provides a qualitative and quantitative view of the variation in metabolite composition during flower bud development and can be used to inform the industry of the best harvesting practices for high-quality FFB-SJ.

## Materials and methods

### Chemicals and reagents

Acetonitrile, methanol, acetic acid, and ethanol were chromatographically graded and were obtained from Merck KGaA (Darmstadt, Germany). Deionized water was obtained using a Milli-Q system (Millipore, Burlington, MA, United States).

### Plant material

**Figure 1:**
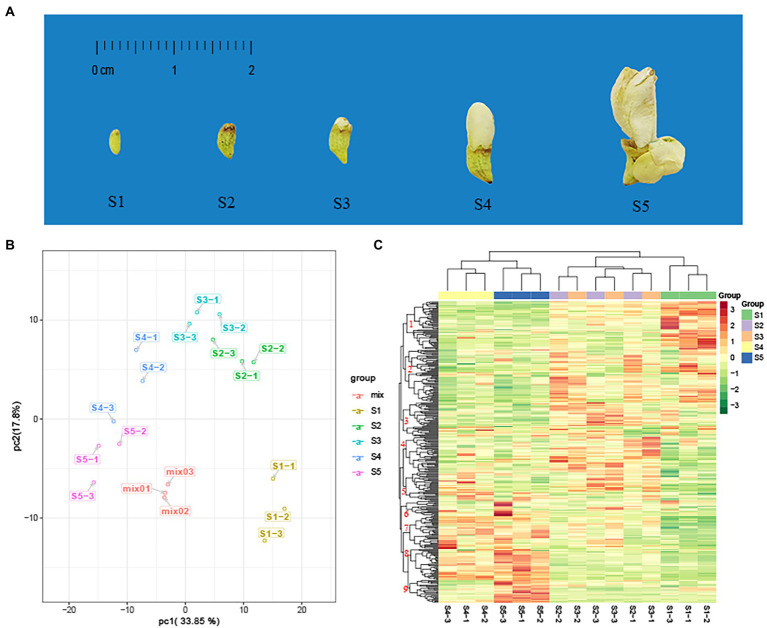
Principal component analysis (PCA) and heat map analysis of metabolites in five developmental stages of the flower of *Sophora japonica*. **(A)** Phenotypic features of *S. japonica* in the five developmental stages of the flower (S1–S5). **(B)** PCA score plot. **(C)** Clustered heat map of all metabolites. A column represents a sample, and a row represents each metabolite. The abundance of each metabolite is represented by a bar with a specific color; red indicates high abundance, whereas metabolites with a low relative abundance are shown in green (color key scale is right of the heat map).

FFB–SJ were collected from the Da Zu district (29°56′N, 105°68′E), Chongqing, China, at an altitude of 379 m. According to their appearance and color, flowers were divided into five developmental stages (S1–S5); the classification criteria are described in our previous article (Wang et al., 2019). The appearance of the FFB–SJ at different growth stages is shown in Figure 1A. Three biological replicates were examined for each stage, and each replicate comprised at least 100 flowers collected from one tree. All samples were harvested on the same day. All materials were washed with distilled water, frozen in liquid nitrogen, and stored at −80°C.

### Extraction

The samples were freeze-dried and crushed using a mixer mill (MM 400, Retsch GmbH, Haan, Germany) with zirconia bead for 1.5 min at 30 Hz. We extracted 100 mg of dry powder from each sample overnight at 4°C using a tenfold (1.0 ml) volume of 70% ethanol. The flower residues were removed by centrifugation at 10,000 × *g* for 10 min, and the supernatants were cleaned by solid-phase extraction (CNWBOND Carbon-GCB SPE Cartridge, 250 mg, 3 ml; ANPEL, Shanghai, China) and filtered before UPLC–MS/MS analysis.

### UPLC and ESI–QTRAP–MS/MS conditions

To detect secondary metabolites in the FFB–SJ extract, we injected 5 μl of the working solution into a UPLC system (Shim-pack UFLC SHIMADZU CBM30A, SHIMADZU, Kyoto, Japan) coupled with an API 4500 Q TRAP (AB Sciex, Framingham, MA, United States). A C18 reversed-phase column (2.1 × 100 mm, 1.8 μm; Acquity UPLC HSS T3 C18, Waters Corporation, Milford, MA, United States) was used for the stationary phase at 40°C. An acetonitrile aqueous solution containing 0.04% (v/v) acetic acid (A) and 0.04% (v/v) aqueous acetic acid (B) were used as the mobile phase at a flow rate of 0.4 ml/min. Gradient elution was performed as follows: 0 min, 0% A; 0–11.0 min, 0–95% A; 11.0–12.0 min, 95% A; 12.0–12.1 min, 95–5% A; and 12.1–15.0 min, 5% A. Secondary metabolites were additionally detected using an API 4500 Q TRAP LC/MS/MS system equipped with linear ion trap (LIT) and triple quadrupole (QQQ) scans. The ESI source was operated as follows: collision activation parameter, 6; air curtain gas, 25 psi; atomization gas (GS1), 55 psi; auxiliary gas (GS2), 60 psi; ion spray voltage (IS), −5,500 V; ion source temperature (TEM), 550°C. Positive and negative ion multi-reaction monitoring (MRM) mode with collision gas (nitrogen, 5 psi) was used for the QQQ scan. A specific set of MRM transitions was monitored based on the metabolites at each elution stage. The declustering potential (DP) and collision energy (CE) were determined through optimization.

### Qualitative and quantitative analysis of metabolites

Qualitative primary and secondary mass spectrometry detection was performed using public and in-house databases of metabolite information (MetWare Biological Science and Technology, Wuhan, China).^1^ In the analysis of metabolite structure, we referred to the Mass Bank,^2^ KNAPSAcK,^3^ HMDB,^4^ METLIN,^5^ and MoTo DB^6^ public databases. Quantitative analysis of metabolites was performed using QQQ MRM mode analysis. After obtaining the spectrum data for all samples, the mass spectrum peaks were integrated for all metabolites and those for the same metabolite were integrated and corrected (Fraga et al., 2010). Metabolite content was expressed as chromatographic peak area integrals.

### Statistical analysis

All data are log_2_-transformed and reported as the mean ± standard deviation of three independent experiments. Mass spectral data were processed using Analyst 1.6.3 software (AB Sciex, Framingham, MA, United States). The differences in metabolite composition from S1–S5 were analyzed using hierarchical clustering analysis (HCA), principal component analysis (PCA), orthogonal partial least squares discriminant analysis (OPLS-DA), and K-means cluster analysis with R 3.5.1 software (The R Foundation, Vienna, Austria). Differential metabolites were screened based on variable influences in projection (VIP) and fold change (FC) values. To determine the corresponding metabolic pathways, differential metabolites were annotated using the Kyoto Encyclopedia of Genes and Genomes (KEGG^7^) database. The network of differential metabolites and pathways was constructed using the Cytoscape 3.80 software (Cytoscape, Boston, MA, United States).

## Results

### Mass spectrometry of secondary metabolites

The total ion current (TIC) plots and multi-peak detection plots of one quality control (QC) sample are shown in Supplementary Figure S1. Overlay analysis of the QC TIC and sample multi-peak detection diagrams (Supplementary Figure S2) showed good repeatability and reliability of the data. A total of 331 secondary metabolites were detected (Supplementary Table S1; Supplementary Figure S3), including 173 flavonoids, 53 phenolic acids, 29 organic acids, 23 alkaloids, 15 terpenes, 11 lignans, coumarins, six tannins, and 21 other metabolites.

### Multivariate analysis revealed differences in metabolite composition

#### PCA and HCA

PCA converts a multidimensional variable system to a low-dimensional variable system with higher accuracy. A principal component score map was used to determine the distribution of each sample to simplify its classification. Based on the UPLC–ESI–QTRAP–MS/MS data, we identified two principal components (PC1 and PC2), reflecting a total variance of 33.85 and 17.80%, respectively. Metabolite composition changed significantly between S1 and S5 (Figure 1B). The data for the three biological replicates of samples S2 and S3 were closely distributed, suggesting that their metabolites were similar. The data are easily distinguishable from the other consecutive stages.

According to the HCA, the replicates for the S1, S4, and S5 samples were grouped into one category on the abscissa axis (Figure 1C). The classification results were highly correlated with the degree of phenotypic differences between developmental stages. The main difference in morphology between the S2 and S3 samples was the appearance of small petals on the top of the flower bud (Wang et al., 2019). Nine major categories were identified on the ordinate axis, according to the accumulation of secondary metabolites. The metabolites in category 1 accumulated at the highest levels in S1, followed by S2 and S3, and at the lowest levels in S4 and S5. Metabolites in category 2 accumulated at the highest levels in S1, S2, and S3 and the lowest levels in S4 and S5. Metabolites in categories 7 and 8 accumulated at the highest levels in S4 and S5, and the lowest levels in S1, S2, and S3. Metabolites in category 9 showed significant accumulation in S5, whereas metabolites in categories 3 and 4 showed more accumulation in S2 and S3.

PCA and HCA results showed that the changes in secondary metabolites during flower maturation could be roughly divided into three major stages (S1, S2–S3, and S4–S5).

#### OPLS-DA

**Figure 2:**
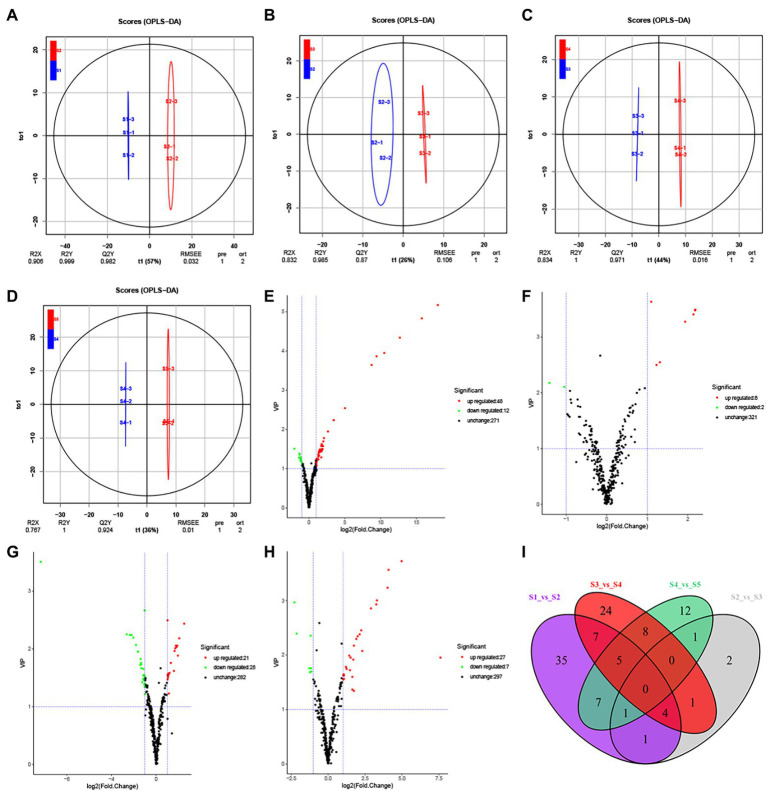
Differential secondary metabolite analysis of the samples in five developmental stages of the *S. japonica* flower. **(A−D)** Orthogonal partial least squares discriminant analysis (OPLS-DA) model plots for the following comparisons: S1 vs. S2, S2 vs. S3, S3 vs. S4, and S4 vs. S5, respectively. **(E−H)** Volcano plots showing the expression levels of the differential secondary metabolites in the comparisons S1 vs. S2, S2 vs. S3, S3 vs. S4, and S4 vs. S5, respectively. Red dots indicate upregulated, differentially expressed metabolites; green dots indicate downregulated, differentially expressed metabolites; and black dots indicate detected metabolites with insignificant differences in expression. **(I)** The Venn diagram indicates the common and unique metabolites in the comparison groups.

We used OPLS-DA models to further compare the differences between the groups (Figures 2A–D; Supplementary Figures S4A–F). The Q2 value of all models exceeded 0.8 (S1 vs. S2, 0.982; S2 vs. S3, 0.870; S3 vs. S4, 0.971; S4 vs. S5, 0.924; S1 vs. S3, 0.987; S1 vs. S4, 0.992; S1 vs. S5, 0.994; S2 vs. S4, 0.987; S2 vs. S5, 0.992; S3 vs. S5, 0.986), indicating suitable model reliability. According to the direction of the abscissa, the samples from consecutive and non-consecutive stages were clearly separated, indicating that the metabolite composition changed significantly during the five stages of development. Contrary to the results of PCA and HCA, the S2 and S3 samples were separated in the OPLS-DA model.

### Differential metabolite screening

Differential metabolites between groups were screened based on FC (FC ≥ 2 or FC ≤ 0.5) and VIP (VIP ≥ 1) values (Figures 2E–I; Supplementary Figures S4G–M). A total of 164 differential metabolites were identified across developmental stages S1–S5 and were distributed across eight categories (Supplementary Table S2). We identified 60 differential metabolites between S1 and S2 (48 upregulated and 12 downregulated), 10 between S2 and S3 (8 upregulated and 2 downregulated), 49 between S3 and S4 (21 upregulated and 28 downregulated), and 34 between S4 and S5 (27 upregulated and seven downregulated). Unlike the unique differential metabolites found in each comparison (Figure 2I), we did not identify any common differential metabolites between the five consecutive stages, indicating that different secondary metabolites participate throughout flower maturation.

Regarding non-consecutive developmental stages, we identified 84 differential metabolites between S1 and S3 (62 upregulated and 22 downregulated), 101 between S1 and S4 (65 upregulated and 36 downregulated), 102 between S1 and S5 (69 upregulated and 33 downregulated), 71 between S2 and S4 (29 upregulated and 42 downregulated), 94 between S2 and S5 (52 upregulated and 42 downregulated), and 81 between S3 and S5 (45 upregulated and 36 downregulated). As the interval between the growth periods increased, the number of differential metabolites increased. A total of 35 differential metabolites were common to S1 and S2, S1 and S3, S1 and S4, and S1 and S5. Meanwhile, 2, 8, 4, and 17 unique differential metabolites were identified in the same comparisons (Supplementary Figure S4M).

### Dynamics of the different metabolites during flower maturation

**Figure 3:**
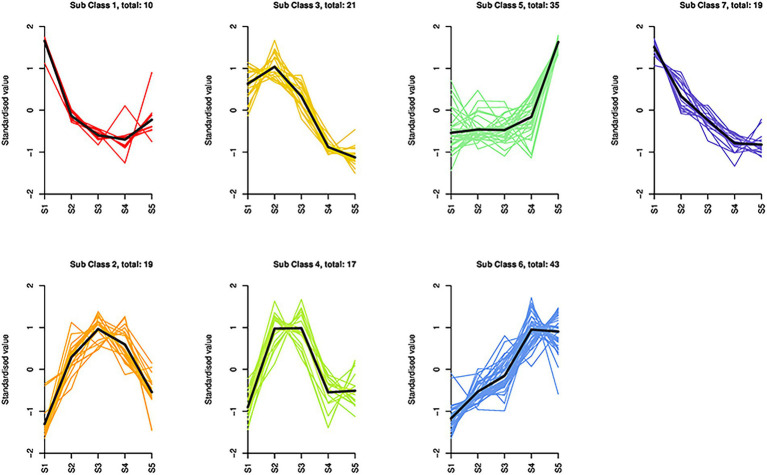
Dynamics of the differential metabolites in *S. japonica* flowers during maturation. Based on K-means cluster analysis, 164 differential metabolites were divided into seven subclasses containing 10, 19, 21, 17, 35, 43, and 19 metabolites.

Based on the K-means cluster analysis, the 164 differential metabolites were divided into seven subclasses containing 10, 19, 21, 17, 35, 43, and 19 metabolites (Figure 3; Supplementary Table S3). Subclasses 1, 3, and 7 showed downward trends from S1 to S5, whereas subclasses 5 and 6 showed upward trends from S1 to S5. In subclasses 2, 3, and 4, the metabolites first increased and then decreased, and the cumulative amount was the highest at S2 or S3. From S3 to S5, 88 metabolites from subclasses 1, 5, and 6 were upregulated, including all anthocyanins (5/5), lignans (3/3), most organic acids (12/16), and flavonols (16/19). All coumarins (2/2) and proanthocyanidins (6/6) were downregulated. The regularity of other types of compounds was not obvious.

### Differential metabolic pathways among the S1–S5 samples

**Figure 4:**
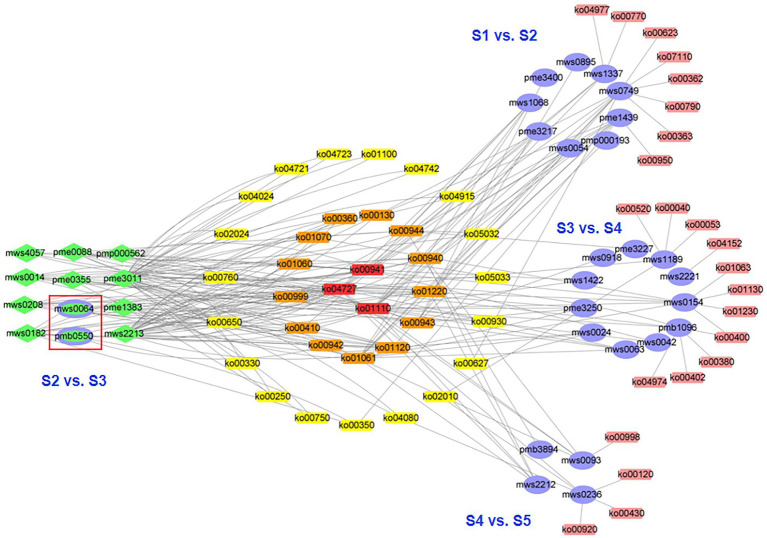
Network visualization of the differential metabolites and metabolic pathways during five developmental stages of the flowers of *S. japonica*. The purple ovals represent the unique differential metabolites for each comparison, green diamonds or ellipses represent the differential metabolites corresponding with multiple comparisons, and green-filled ovals (purple border) represent the differential metabolites for the comparison of stages 2 and 3 (S2 vs. S3).

Between consecutive stages, 36 differential metabolites were distributed across 58 pathways (Figure 4), 17 differential metabolites between S1 and S2 were distributed in 27 pathways, 2 between S2 and S3 were distributed in 4 pathways, 19 between S3 and S4 were distributed in 45 pathways, and 13 between S4 and S5 were distributed in 36 pathways (Supplementary Table S4). Regarding samples from nonconsecutive stages, we identified 20 additional metabolic pathways, 15 of which were marked from S1 to S4, four from S1 to S5, one from S2 to S4, four from S2 to S5, and three from S3 to S5. No additional metabolic pathways were identified in S1 to S3 (Supplementary Table S4).

Three metabolic pathways were involved in four consecutive stages (ko01110, biosynthesis of secondary metabolites; ko00941, flavonoid biosynthesis; and ko01100, metabolic pathways; Figure 4), indicating that these metabolic pathways were active and played important roles throughout flower maturation. In particular, 23 metabolic pathways were identified between consecutive stages (8, S1 vs. S2; 0, S2 vs. S3; 11, S3 vs. S4; 4, S4 vs. S5). These pathways could be used as markers for studying phenotypic development in FFB-SJ. Notably, metabolic pathways related to the biosynthesis of flavonoids, including “anthocyanin biosynthesis,” “flavonoid biosynthesis,” and “isoflavonoid biosynthesis,” were significantly enriched (*p* < 0.05) in the early stages of flower development (S1 to S3; Supplementary Figures S5A,B). From S3 to S4, the differential metabolites were enriched in the pathways for “phenylalanine, tyrosine, and tryptophan biosynthesis,” “phenylalanine metabolism,” “microbial metabolism in diverse environments,” and “biosynthesis of plant hormones” (Supplementary Figure S5C). In S4–S5, the differential metabolites were enriched in the “microbial metabolism in diverse environments” pathway as well as pathways related to chemical structure transformation, such as “phenylpropanoid biosynthesis,” “metabolic pathways,” and “degradation of aromatic compounds” (Supplementary Figure S5D). From S3 to S5, metabolite pathways related to flower maturation, including “GABA-ergic synapse,” “cAMP signaling pathway,” “estrogen signaling pathway,” and “biosynthesis of plant hormones,” were significantly enriched (*p* < 0.05).

## Discussion

The quality of FFB–SJ is closely related to its chemical components, the composition of which varies with flower maturity. During plant growth, the accumulation of secondary metabolites is determined by the environment, which in turn affects the value of raw materials. We propose that by elucidating the development and metabolism of *S. japonica* one could determine the mechanisms affecting the quality of FFB-SJ and inform resource utilization.

We identified 164 differential metabolites among the different growth stages of *S. japonica*. When comparing consecutive stages, S1 and S2 showed the largest difference in metabolite composition, reflected in the number of metabolites per type, indicating that metabolism plays an important role in the early stages of flower bud development. Although the differences in phenotype during this period were mainly related to the size of the flower buds, many flavonoids and phenolic acids were also upregulated (Supplementary Table S2). The most common secondary metabolites in FFB–SJ are flavonoids, which protect young organs from environmental stresses (Shah and Smith, 2020). Phenolic acids are directly involved in plant growth regulation (Yu et al., 2021a) and are important precursors of flavonoid synthesis (Chen et al., 2021). Both flavonoids and phenolic acids are used in clinical applications in humans (Sharma et al., 2020). A total of 37 flavonoids or phenolic acids showed the highest accumulation at S2 and S3 (included in sub-classes 2, 3, and 4). Most of these components have positive therapeutic effects on cardiovascular and hepatic diseases. For example, genistein (El-Far et al., 2022), epigallocatechin gallate (George et al., 2022), pinocembrin (Cao et al., 2022), and p-coumaric acid (Bal et al., 2022) of *Flos Sophorae Immaturus* are effective in treating liver-related diseases, clearing the liver, and ameliorating hepatic fire. Genistin (Gu et al., 2016), catechin (Rodriguez et al., 2006), and cinnamic acid (Luan et al., 2022) complement the treatment of cardiovascular diseases by cooling the blood and inhibiting hemorrhage. From S2 to S3, most metabolites remained unchanged, whereas anthocyanins were upregulated. The change in anthocyanins is mainly related to the composition of the petal color, with the petals gradually sprouting from the top of the calyx during this period (Figure 1A).

During the middle and late stages of flower development (S3–S5), the flower phenotype changed considerably. During this period, the upregulation of anthocyanins and downregulation of proanthocyanidins generates the beautiful colors of *S. japonica* flowers (Guo et al., 2022). The vast majority of differential organic acids and flavonols were upregulated (Figure 3; Supplementary Table S3). Some organic acids participate in regulating flower development. For example, γ-aminobutyric acid, which was upregulated in S3–S5 samples, promotes pollen tube formation and regulates the opening and closing of the corolla (Yu et al., 2014). Shikimic acid and 2-methylglutaric acid are associated with the flower phenotype of *S. japonica*; these metabolites are intermediate substances in the metabolic pathways of biological regulatory processes (Yokoyama et al., 2021). Owing to the absence of a large substituent at the 3-hydroxyl group, the 4-carbonyl-5 hydroxyl structure of flavonols can easily bind to enzymes to promote antioxidant and anti-tyrosinase activity (Xue et al., 2011). In addition, phenolamines were downregulated, which improved overall taste.

As the flower matures, FFB-SJ yield increases. The dried weight of 1,000 grains of FFB–SJ varied significantly among the five stages (Supplementary Table S5), with the highest yields observed at S4–S5. Quality and yield are important indicators of plant value, particularly in the medical and food industries. Our previous study showed that the total flavonoid content from S1 to S3 was higher than that in S4 and S5 (Wang et al., 2019), but S1 buds generated far lower yields than S2 and S3 (Supplementary Table S5). In S2 and S3, the flower buds expanded and became rich in flavonoids; thus, these buds are suitable for use in high-quality medicines or industrial extraction of flavonoids. Indeed, *S. japonica* should be harvested when it is immature (Liao and Cheng, 2012). Compared with the bud (S2–S3), the fully developed flower (S4–S5) has obvious advantages in terms of yield, but the total flavonoid content and biological activities are diminished (Cholak et al., 2016; Wang et al., 2019).

## Conclusion

Due to its quality, *S. japonica* has attracted increasing attention as a raw material for medicine and food. We used targeted metabolomics technology to analyze secondary metabolites in *S. japonica* during the five stages of flower maturation. We monitored 331 metabolites and screened 164 differential metabolites that showed seven distinct trends between the stages of development. Polyphenols, including flavonoids and phenolic acids, are major metabolites. The variation in these metabolites corresponds to the developmental requirements of *S. japonica* and affects the quality of FFB–SJ as a medicinal and industrial material. Flower buds (S2–S3) contain a rich variety of polyphenols; the high total flavonoid content at this stage increases the medicinal and industrial value of the buds. Although we examined trends in metabolite composition, the mechanisms underlying this variation remain unclear. In future studies, multi-omics analysis should be employed to determine the regulatory mechanisms of these metabolites and how they respond to varying environmental conditions.

## Data availability statement

The original contributions presented in the study are included in the article/Supplementary material, further inquiries can be directed to the corresponding author.

## Author contributions

J-RW: Conceptualization, resources, visualization, writing—review, and editing. X-HS: Investigation and resources. L-YL: Methodology, supervision, project administration, and funding acquisition. S-JG: Visualization. F-HS: Software. X-MZ: Writing—review and editing. YY: Formal analysis. All authors have contributed to the manuscript and approved the submitted version.

## Funding

Financial support was provided by the Natural Science Foundation Project of Chongqing (cstc2020jcyj-msxmX0828), the Basic Research Projects of Chongqing City (jxjl20210001), National Technical System of the Chinese Medicinal Materials Industry (CARS-21), and the Technical System of the TCM Industry in Chongqing [2021(10)].

## Conflict of interest

The authors declare that the research was conducted in the absence of any commercial or financial relationships that could be construed as a potential conflict of interest.

## Publisher’s note

All claims expressed in this article are solely those of the authors and do not necessarily represent those of their affiliated organizations, or those of the publisher, the editors and the reviewers. Any product that may be evaluated in this article, or claim that may be made by its manufacturer, is not guaranteed or endorsed by the publisher.

## Supplementary material

The Supplementary material for this article can be found online at: https://www.frontiersin.org/articles/10.3389/fpls.2022.916410/full#supplementary-material

## Footnotes

^1^http://www.metware.cn/ (Accessed July 22, 2022).

^2^http://www.massbank.jp/ (Accessed July 22, 2022).

^3^http://kanaya.naist.jp/KNApSAcK/ (Accessed July 22, 2022).

^4^http://www.hmdb.ca/ (Accessed July 22, 2022).

^5^http://metlin.scripps.edu/index.php (Accessed July 22, 2022).

^6^http://www.ab.wur.nl/moto/ (Accessed July 22, 2022).

^7^http://www.genome.jp/kegg/ (Accessed July 22, 2022).
